# Public health response and lessons learned from the 2014 chikungunya epidemic in Grenada

**DOI:** 10.26633/RPSP.2017.57

**Published:** 2017-06-30

**Authors:** Martin S Forde, Francis Martin, George Mitchell, Satesh Bidaisee

**Affiliations:** 1 Public Health & Preventive Medicine Public Health & Preventive Medicine St. George Grenada Public Health & Preventive Medicine, St. George’s University, St. George, Grenada.; 2 Ministry of Health Ministry of Health St. George Grenada Ministry of Health, St. George, Grenada.

**Keywords:** Chikungunya virus, Grenada, Caribbean region, Virus chikungunya, Grenada, Región del Caribe

## Abstract

In June 2014, the first cases of chikungunya virus (CHIKV) were diagnosed on the island of Carriacou, part of the tri-island state of Grenada. In the three months that followed, CHIKV spread rapidly, with conservative estimates of the population infected of at least 60%. Multiple challenges were encountered in the battle to manage the spread and impact of this high–attack rate virus, including 1) limited indigenous laboratory diagnostic capabilities; 2) an under-resourced health care system; 3) a skeptical general public, hesitant to accept facts about the origin and mode of transmission of the new virus; and 4) resistance to the vector control strategies used. Lessons learned from the outbreak included the need for 1) a robust and reliable epidemiological surveillance system; 2) effective strategies for communicating with the general population; 3) exploration of other methods of mosquito vector control; and 4) a careful review of all health care policies and protocols to ensure that effective, organized responses are triggered when an infectious outbreak occurs.

## RESUMEN

En junio del 2014, se diagnosticaron los primeros casos de infección por el virus del chikungunya (CHIKV) en la isla de Carriacou, perteneciente al Estado triinsular de Granada. En los tres meses siguientes, el CHIKV se diseminó con rapidez, llegando a afectar, según cálculos moderados, al menos al 60% de la población. Se encontraron varias dificultades en la batalla por controlar la propagación y las consecuencias de este virus que tiene una alta tasa de ataque, a saber: 1) limitaciones de la capacidad de diagnóstico de los laboratorios locales; 2) escasez de recursos del sistema de atención de salud; 3) escepticismo general del público, reacio a aceptar los datos sobre el origen y la vía de transmisión del nuevo virus; y 4) resistencia a las estrategias empleadas para controlar los vectores. Entre las enseñanzas extraídas del brote cabe señalar la necesidad de lo siguiente: 1) un sistema sólido y confiable de vigilancia epidemiológica; 2) estrategias eficaces para comunicarse con la población general; 3) exploración de otros métodos de control de mosquitos vectores; y 4) un examen meticuloso de todas las políticas y protocolos de atención de salud para garantizar que se activen respuestas eficaces y organizadas cuando se produce un brote infeccioso.

Since the first laboratory-confirmed case of chikungunya virus (CHIKV) in Saint Martin in December 2013, the mosquito-borne viral pathogen has spread rapidly throughout the Caribbean chain of islands (1, 2). In June 2014, the first cases of CHIKV infection were diagnosed in the tri-island state of Grenada, Carriacou, and Petit Martinique. The virus spread rapidly, with conservative estimates of as much as 60% of the population infected in just three months of intense transmission (3). The epidemic was attributed to CHIKV, rather than the dengue virus (DENV), as CHIKV was the prevailing regional burden reported for the Caribbean region during this period. Epidemiologic investigation of suspected cases occurring after the index case did not reveal any co-morbidity with DENV (4).

## RESPONSE TO CHIKV IN GRENADA

### Pre-epidemic

In March 2014, Grenada’s Ministry of Health (MoH) established a Chikungunya Task Force (CTF) Committee with a mandate to review the country’s ability to preempt, to the greatest extent possible, CHIKV entry into the tri-island state. The Committee was co-chaired by the MoH and the Chief Medical Officer and included key government personnel from the MoH Vector Control Unit (VCU); other senior medical officers and the director of Grenada’s main hospital (General Hospital in St. George); directors from several MoH nursing divisions; the Chief Environmental Health Officer; officials from the ministries of Foreign Affairs and Tourism, Civil Aviation and Culture; the Grenada Solid Waste ManagementAuthority; and several academics from St. George’s University. The Committee focused its efforts on three types of measures: 1) preventive, 2) educational, and 3) preparatory.

#### Preventive measures.

The first tasks undertaken by the CTF Committee was to try and identify where the first cases of CHIKV would likely arise. Based on this analysis it was determined that the first CHIKV cases would most likely come from persons engaged in the fishing industry. Due to frequent trading among fishing communities across the different islands, it was determined that people working in the fishing industry would most likely be the first to be infected by CHIKV and thus bring the virus into the country. Therefore, all major fishing communities in the two northern islands of Carriacou and Petite Martinique were initially targeted to receive informational and educational materials on CHIKV.

The CTF Committee also identified an annual sailing regatta festival, the Bequia Easter Regatta, held from the middle to the end of April 2014, with extensive involvement of the local sailing community from the islands of Carriacou and Petite Martinique, as the most likely time for the first CHIKV cases to appear.

Given that the CHIKV outbreak was spreading at a rate of one new island per week, when the first CHIKV cases were identified in St. Vincent & the Grenadines (the Caribbean islands closest to Grenada in the North), the islands of Carriacou and Petite Martinique were identified as being the most likely to come under immediate threat of infection. One of the Senior Medical Officers in the MoH and the Chief Environmental Health Officer were dispatched to those islands to provide further information to the fishing communities and the general population with regard to their role in helping reduce their risk of getting infected with CHIKV.

Possible preventive measures that could help delay the entry of the virus into the main island of Grenada were also explored. Due to limited resources, it was decided that fogging would be limited to areas previously identified as having high numbers of both mosquitoes and humans.

In addition, several national cleanup campaigns were arranged and promoted by the government to help reduce the number of potential mosquito breeding sites. Throughout the three islands, community-based activities were organized and supported by government personnel to help de-bush areas around peoples’ homes and eliminate potential sources of standing water where mosquitoes could breed.

#### Educational measures.

Along with the preventive measures listed above, a concerted effort was made to develop and distribute educational materials to certain key sectors of the government as well as the wider general public. Flyers and pamphlets on CHIKV were developed for Customs and Immigration Officers and other workers at the island’s various ports of entry. Several CTF Committee members were asked to give presentations to various governmental and nongovernmental organizations working in agricultural, religious, and hotel/tourism sectors.

To properly sensitize and prepare the general population for the arrival of CHIKV, television and radio public service announcements (PSAs) were made and widely broadcasted. A key goal of the PSAs was to help the public understand the central role played by the *Aedes aegypti* mosquito in the spread of CHIKV and identify specific environmental measures that could be carried out around their homes to help mitigate the spread of the virus.

To buttress government efforts to encourage the island’s population to actively support environmental control measures, the CTF Committee also reviewed several already-enacted laws dealing with garbage disposal, vector control, and other public health hazards. Specific PSAs were disseminated to remind the public that the Mosquito Control Regulations under the Public Health Act of the revised laws of Grenada of 1990 and the Litter Control Act of 1958 gave the government’s Environmental Health Officers the power to issue summary fines for any violations of these laws.

In addition, all local doctors were provided with materials to help them recognize the classic symptoms of CHIKV. This guidance helped a physician identify the first CHIKV cases. After observing certain symptoms among members of various fishing communities in Carriacou in midto late May 2014, he collected blood samples and had them analyzed for CHIKV. These tests resulted in the first five laboratory-confirmed cases of CHIKV, during the first week of June 2014. Therefore, Grenada succumbed to the 2014 Caribbean CHIKV outbreak in epidemiological week 23.

#### Preparatory measures.

Although concerted efforts were being made to prevent the entry of CHIKV into Grenada, it was recognized early on that they would, at best, only delay the inevitable arrival of the virus into the tri-island state. Subsequent efforts were therefore focused on 1) appraising the country’s ability to diagnose and treat those infected with CHIKV, followed by 2) review of possible ways in which the spread and transmission of the virus throughout the rest of the population could be slowed.

An inventory check was done at the government Central Medical Stores to ensure an adequate supply of paracetamol (acetaminophen) was on hand. In addition, requests were made to several external agencies for insecticide-treated bed nets, which were then distributed to the island’s six health centers.

Response protocols for health care personnel were also discussed and drawn up. For example, it was decided that all identified CHIKV cases would be quarantined by placing them under bed nets. In addition, the MoH VCU would be notified of the patient’s address and asked to conduct fogging in that person’s local area.

### Mid-epidemic

Once the first cases of CHIKV were detected on the northern island of Carriacou, vector control efforts on the ferry, a primary means of transportation between Carriacou and Grenada, were stepped up.

Guidelines for clinical management and fast-tracking of patients accessing care at all health care facilities were drawn up by the government’s Epidemiology department and distributed to all health care centers. The key objective of the guidelines was to decrease waiting time for treatment of infected persons in order to reduce the risk of cross-infection.

Upon their receipt, the insecticide-treated bed nets requested at the beginning of the outbreak were distributed to the homes of the first persons diagnosed with CHIKV. Identified cases were encouraged to observe quarantine conditions for their own protection as well as that of their family and the community. In addition, community health workers were sent to the local areas of the first CHIKV patients and the surrounding communities and given advice on how to best avoid becoming infected themselves. Infected persons were advised to remain under their bed nets throughout the day, given that the mosquito vector was active during the day.

As the numbers of those presenting with CHIKV rapidly increased, the available supply of bed nets was soon exhausted. To help minimize further transmission of the infection, infected people were encouraged to self-quarantine as best as possible by purchasing bed nets, using protective clothing and indoor insecticide products, placing screens on all windows and doors, and properly covering any water storage containers.

In addition, for the first few confirmed cases of the virus, the MoH VCU was given the patient’s home address and asked to fog the local surroundings. However, when the outbreak spread rapidly throughout the island, the fogging strategy was no longer feasible and was subsequently stopped.

#### Challenges.

There were multiple challenges in trying to manage the spread and impact of the CHIKV epidemic. First, due to a lack of in-country CHIKV laboratory diagnostic capabilities, the samples had to be sent to Trinidad for confirmation, with a turnaround time of approximately two weeks. Given that as many as four people can be infected with CHIKV via one infected person (5), this delay made continued reliance on laboratory confirmation impractical. Therefore, subsequent diagnosis of CHIKV infection primarily relied on clinical evidence alone. Another challenge was the fact that dengue is endemic to Grenada and spread by the same mosquito vector, making a clear differential diagnosis difficult.

Second, the effectiveness of advising persons diagnosed as infected with CHIKV to stay under bed nets is unclear. Due to limitations on the amount of sick leave a person could secure, and the difficulty of staying under a bed net the entire day during the viremic phase to avoid being bitten by the diurnal vector, it is unlikely that many people complied with this control strategy.

Third, the CHIKV epidemic outbreak, like most epidemic outbreaks, had a significant negative impact on Grenada’s medical care infrastructure, and health care provider workforce, which was significantly weakened when manyhealth care providers at Grenada’s General Hospital who contracted CHIKV were unable to provide patient care throughout their infection and convalescence.

There were also some limitations in the effectiveness of the fogging operation in several communities. Due to apparent health concerns, many villagers elected to close their windows and doors rather than allow the fog to penetrate their homes. Insecticides used in fogging are known to have deleterious effects on other organisms, including humans (6). Another barrier to the use of fogging was the widely pervasive view that CHIKV was not spread by mosquitoes but was caused by an airborne pathogen spread by sick persons sneezing or coughing on others.

In addition, even before the 2014 Caribbean CHIKV outbreak had completed its course, the Caribbean region was forced to deal with the then-emerging threat of the 2014 West Africa Ebola crisis. With all efforts turned to the unprecedented West African Ebola outbreak and its threat to Grenada, conducting a comprehensive post-mortem of the CHIKV response was difficult. Since then, given its mode of transmission, and a number of other factors, Ebola has not made an entry into the Caribbean and is unlikely to do so.

### Post-epidemic

Based on data collected from previous CHIKV outbreaks elsewhere, up to 15% of those infected can have prolonged adverse symptomology. A number of clinicians are now reporting the observance of many patients with what is clinically consistent with post-CHIKV sequelae, especially large joint destruction.

After the first CHIKV index case was identified in Grenada in 2014, 493 sera samples were collected (9 July–9 October) (4). Of these samples, 426 (86%) were found to be positive for CHIKV by either IgM or PCR. Cases ranged in age from 1 year old to more than 90 years old, with a median age of 34.5 years. Thirty different symptoms were observed clinically in the cases enrolled in the study. Due to the absence of co-circulating DENV, the study confirmed that the 2014 epidemic was caused almost exclusively by CHIKV.

Efforts are now under way to conduct a comprehensive review of the impacts of the 2014 Caribbean CHIKV outbreak and explore the potential for further study of the epidemiology and pathogen-host interactions of this new virus in the Caribbean (4).

## DISCUSSION

### Lessons learned

Several important lessons have been gleaned from Grenada’s experience with the Caribbean CHIKV epidemic in 2014. First, without severe (and impractical) travel restrictions, it is virtually impossible to prevent the introduction of this type of vector-borne disease into Grenada’s national territory. Once a competent vector is present on the island, without an established vector control, an outbreak is inevitable, despite the best efforts to prevent and control the entry of the virus into the country. Preventive and control strategies should thus be focused primarily on vector control and interventions designed to limit transmission of the virus from infected to uninfected persons.

Another important lesson learned from the 2014 outbreak is the need to try and predict, and then prepare for, skepticism among the general public about the information provided by health authorities about the origin and mode of transmission of a disease that is new to them. The public’s perception of where and how a new disease is spread can have a significant effect on their behavior patterns, including those divergent, and in some cases diametrically opposed, to the desired response. Misinformation and urban myths can gain surprisingly strong traction to the point where they can become immune to even concerted efforts to debunk or derail them. One example of a myth that became entrenched in the minds of many Grenadians was that CHIKV was not mosquito-borne but rather an airborne respiratory pathogen spread by sick persons sneezing or coughing on others. In addition, given the severe arthralgia many CHIKV-infected persons experienced, many so-called natural treatments were promoted with little or no evidence provided about their efficacy or, more important, potential harm. One natural treatment that was heavily recommended at the height of the outbreak and drew much attention from the public was the notion that pouring boiling water on papaya leaves and drinking the resultant brew could cure the disease within 48 hours of the joint pain effects caused by CHIKV infection.

While clinical evidence and epidemiological data were sufficient, under the circumstances, for prescribing appropriate treatments and making decisions about the need for health care resources, the lack of indigenous laboratory capacity was a major impediment in the management of this outbreak. For example, unpublished government data showed a dramatic drop in the number of dengue cases (from 155 in 2013 to 39 in 2014), which may have been due to misclassification of dengue cases as CHIKV cases. The importance of establishing a clear case definition to help clinicians differentiate dengue virus infection from CHIKV infection, and thus allow for accurate monitoring of the emerging outbreak by health authorities, was demonstrated during the CHIKV outbreak in Saint Martin (7). Having differential diagnoses for the two diseases is important because treatment protocols, along with disease management, and outcomes, are slightly different. For example, adverse health effects for dengue are more severe than those for CHIKV, and potentially life-threatening.

The limited resources available to the government for providing beds nets population-wide underscores the need for continued, focused efforts to control the vector to help prevent disease transmission. In addition to fogging, interventions aimed at reducing egg and larva development should be key components in the fight to reduce the mosquito population. Community engagement on proper management of the environment must be developed and sustained, nationwide, throughout both the wet and dry seasons, to help prevent the number of habitats in which mosquitoes can breed and thrive.

Finally, in order to implement effective preventive measures, a robust and reliable epidemiological surveillance system is essential. For those directly involved in the management of the outbreak, accurate and timely incidence data are needed to evaluate the efficacy of any measures taken to halt or slow the transmission of the virus throughout the population (8). If the surveillance system can be designed to capture geo-referencing data, as was the case in Dominica, the clusters can be identified more dynamically, which can in turn facilitate community-level assessment of risk and provide opportunities for targeted control efforts (9). Accurate and readily available data can also enable others, such as those engaged in the tourism industry, to properly advise prospective tourists in determining the level of safety against potential disease transmission. This is of particular importance to Grenada, given the heavy dependence of the country’s economy on revenue earned from the tourism industry.

### Limitations

The overall utility of the lessons learned from the 2014 mosquito-borne outbreak of CHIKV in Grenada was limited by the fact that the public health response was reactive rather than planned with a view to determining which interventions would have the best material impact. Due to this limitation, plausible conclusions about the effectiveness of certain preventive actions cannot be made from reviewing the experience of Grenada public health officials during the epidemic. For example, the effectiveness of using bed nets to minimize the further spread of the virus cannot be compared to that of increased fogging or other control strategies directed at the vector. The 2014 outbreak does, however, highlight the barriers and challenges typically faced by many health care systems operating in resource-limited settings and can thus help authorities vested with the role of protecting public health in planning interventions to manage epidemics.

## Conclusions

Reflection on the challenges and lessons learned from the 2014 Caribbean CHIKV outbreak has helped the Grenadian government determine how they might have approached that outbreak or future outbreaks differently. For example, the CHIKV outbreak continues to highlight problems associated with the use of fogging as a prime mosquito control strategy. In addition, there is growing resistance to the use of fogging, further limiting the effectiveness of this vector control method, as people try their best to prevent the insecticidal fog from infiltrating their homes. In addition, efforts to enforce current laws that deal with garbage disposal, vector control, and other public health hazards must be increased upstream. Finally, given the possibility that misinformation and myths will emerge, particularly with the emergence of new diseases, public education efforts should be carefully thought out, targeted, and community-derived. Finally, high–attack rate infections such as CHIKV highlight the necessity for countries to carefully review and implement any necessary changes and improvements in their health care policies and infrastructures so that 1) appropriate, organized responses are triggered when the outbreak occurs, and 2) effective communication with the population at large is achieved.

## Disclaimer.

Authors hold sole responsibility for the views expressed in the manuscript, which may not necessarily reflect the opinion or policy of the *RPSP/PAJPH* or the Pan American Health Organization (PAHO).

## References

[1] 1. Centers for Disease Control and Prevention. Update on emerging infections: news from the Centers for Disease Control and Prevention. Notes from the field: chikungunya virus spreads in the AmericasCaribbean and South America, 2013–2014. Ann Emerg Med. 2014;64(5):552-3. 10.1016/j.annemergmed.2014.08.002 25640241

[2] 2. Morrison TE. Reemergence of chikungunya virus. J Virol. 2014;88(20): 11644-7. 10.1128/JVI.01432-14 PMC4178719 25078691

[3] 3. Jungkind K, Yearwood K, Mitchell G, David-Antoine A, Macpherson CNL, Noel TP, et al. Lessons learnt from the intense 2014 chikungunya epidemic inGrenada. West Indian Med J. 2015;64 (Suppl 2):86.

[4] 4. LaBeaud AD, Heath CJ, Noël TP, Jungkind D, Mitchell G, Yearwood K, et al. Chikungunya in the western hemisphere: a review of the 2014 epidemic, the potential long-term impact, and research opportunities. West Indian Med J. 2015;64 (Suppl 2):31.

[5] 5. Cauchemez S, Ledrans M, Poletto C, Quenel P, de Valk H, Colizza V, et al. Local and regional spread of chikungunya fever in the Americas. Euro Surveill. 2014;19(28): 20854. 10.2807/1560-7917.es2014.19.28.20854 PMC4340072 25060573

[6] 6. Flessel P, Quintana PJ, Hooper K. Genetic toxicity of malathion: a review. Environ Mol Mutagen. 1993;22(1):7-17. 10.1002/em.2850220104 8339727

[7] 7. Omarjee R, Prat C, Flusin O, Boucau S, Tenebray B, Merle O, et al. Importance of case definition to monitor ongoing outbreak of chikungunya virus on a background of actively circulating dengue virus, St Martin, December 2013 to January 2014. Euro Surveill. 2014;19(13). pii: 20753. 10.2807/1560-7917.es2014.19.13.20753 24721537

[8] 8. Mowatt L, Jackson ST. Chikungunya in the Caribbean: an epidemic in the making. Infect Dis Ther. 2014;3(2):63-8. 10.1007/s40121-014-0043-9 PMC4269617 25245516

[9] 9. Nsoesie EO, Ricketts RP, Brown HE, Fish D, Durham DP, Ndeffo Mbah ML, et al. Spatial and temporal clustering of chikungunya virus transmission in Dominica. PLoS Negl Trop Dis. 2015;9(8): e0003977. 10.1371/journal.pntd.0003977 PMC4537218 26274813

